# Robots with tears can convey enhanced sadness and elicit support intentions

**DOI:** 10.3389/frobt.2023.1121624

**Published:** 2023-06-01

**Authors:** Akiko Yasuhara, Takuma Takehara

**Affiliations:** ^1^ Graduate School of Psychology, Doshisha University, Kyotanabe, Kyoto, Japan; ^2^ Department of Psychology, Doshisha University, Kyotanabe, Kyoto, Japan

**Keywords:** human-robot interaction, communicative robot, social robot, emotional tears, sadness, crying

## Abstract

The behaviour of shedding tears is a unique human expression of emotion. Human tears have an emotional signalling function that conveys sadness and a social signalling function that elicits support intention from others. The present study aimed to clarify whether the tears of robots have the same emotional and social signalling functions as human tears, using methods employed in previous studies conducted on human tears. Tear processing was applied to robot pictures to create pictures with and without tears, which were used as visual stimuli. In Study 1, the participants viewed pictures of robots with and without tears and rated the intensity of the emotion experienced by the robot in the picture. The results showed that adding tears to a robot’s picture significantly increased the rated intensity of sadness. Study 2 measured support intentions towards a robot by presenting a robot’s picture with a scenario. The results showed that adding tears to the robot’s picture also increased the support intentions indicating that robot tears have emotional and social signalling functions similar to those of human tears.

## 1 Introduction

The ability of communicative robots to express emotions in various ways is critical for human-robot interaction. People communicate with each other using language and nonverbal cues such as gestures and facial expressions to convey their emotions and infer others’ emotions (Barrett, 1993; Lange et al., 2022). By expressing emotions in the same manner as humans, the signals and intentions sent by robots can be intuitively understood by humans, without these having to undergo special training for interaction with robots. Thus, it is essential for robots to express emotions to facilitate human-robot interactions (Cañamero and Fredslund, 2001; Eyssel et al., 2010; Kim and Kwon, 2010; Złotowski et al., 2015; Tsiourti et al., 2019). Furthermore, it has been argued that emotions have a social function (van Kleef and Côté, 2022). the social functionof emotions refers to the influence of emotional expressions on the thoughts, feelings, and behaviours of others, which has been widely confirmed in the emotional expressions of robots (Melo et al., 2023). For example, it has been demonstrated that when robots express emotions through language, gestures, and facial expressions, they increase their ratings of intelligence, sociability (Garrell et al., 2017), humanlikeness, likeability, and closeness (Eyseel et al., 2010). In addition, field research in supermarkets has shown that children are interested in robots that exhibit emotional expressions and engage with them themselves (Motoda, 2019). In short, the social function of emotions has many advantages, as it works not only between people but also between people and robots (Melo et al., 2023). Additionally, many studies have shown that emotional expressions by robots can change peoples’ attitudes towards them and influence their emotions and behaviours (e.g., Garrell et al., 2017; Motoda, 2019). Moreover, Cañamero and Fredslund. (2001) argued that more emotional expressions are required for richer human-robot interactions. In addition, robots should be able to express diverse and complex emotions that are similar to those of humans.

In September 2021, a robot that sheds tears like a human was developed at a University in Japan. The robot has tear sacs in its eyes, moistens its eyes, and sheds tears naturally (Yoon, 2021). Associate Professor Sejima, the robot developer, hopes to create an atmosphere that allows people to cry easily in the presence of crying robots. The robot is intended for tele-counselling and activities that aim to relieve stress by crying (Yoon, 2021).

However, it is unclear whether the tears shed by robots are recognised as emotional expressions. The behaviour of shedding tears in response to emotional arousal is unique to humans (Vingerhoets, 2013). In studies on non-human subjects, the tears of animals and avatar faces have been found to convey sadness (Gračanin et al., 2021; Picó and Gadea, 2021). However, to the best of our knowledge, no studies have examined people’s perceptions of tears shed by robots. In other words, it is not clear whether the social functions of emotions that arise from people shedding tears work in the same manner when robots shed tears. Therefore, even if a robot is equipped with the ability to shed tears, it is unclear whether it will have the expected effects such as emotional communication and interaction. Hence, it is essential to clarify this issue when designing future robots. As a result, the present study aimed to determine whether robot tears also exhibit the typical signalling functions of human tears using methods employed in previous studies conducted on human tears. In the next section, the typical emotional and social signalling functions of human tears are reviewed. Finally, the overview and hypotheses of the present study are presented.

Human tears function as a signal that conveys sadness. Provine et al. (2009) and Zeifman and Brown (2011) showed that removing tears from a picture of a tearful person reduced the intensity ratings of sadness. Conversely, adding tears to a sad expression accelerates the rate at which it is perceived as sad, indicating that tears facilitate the perception of sadness (Balsters et al., 2013). Ito et al. (2019) demonstrated that when tears were added to neutral, sad, angry, fearful, and disgusted facial expressions, the rated intensity of sadness was higher for all expressions and the rating pattern of facial expressions approached that of sadness. They concluded that tears are convey sadness.

Additionally, tears can function as a social signal to elicit support intentions from others. Several studies examined the effect of tears on eliciting support intentions using pictures of tearful people or digitally manipulated pictures of tears. The results have consistently shown enhanced support intentions for tearful individuals compared to those who are not tearful (Hendriks and Vingerhoets, 2006; Vingerhoets et al., 2016; Stadel et al., 2019). The effect of tears on eliciting support intentions from others was proposed by Zickfeld et al. (2021) as a social support hypothesis. They demonstrated that tears elicit support intention from others; however, the magnitude of the effect of tears varies depending on certain variables such as the event of crying, relationship with the person, and gender. Recently, the process by which tears elicit support intentions has become increasingly apparent. Vingerhoets et al. (2016) and Zickfeld et al. (2021) demonstrated that tears represent feelings of helplessness and warmth. Observers feel connected to a tearful individual, which leads to supporting intentions towards the latter.

## 2 Hypotheses and overview of the study

This study investigated whether tears shed by robots have both emotional and social signalling functions, using methods similar to those employed in previous studies conducted on human tears. Specifically, we used pictures of robots as visual stimuli and measured self-reported responses. These responses provide the first clue to people’s feelings and behaviours in real-life situations. In other words, we aimed to determine whether the responses to a robot’s tears were similar to those to human tears. Study 1 investigated whether robot tears serve as emotional signals that convey sadness. Study 2 investigated whether robot tears served as a social signal that elicits support intentions. The process underlying the effect of human tears on support intentions has been identified in existing literature; therefore, Study 2 also examined whether robots and humans were similar in this process.

Because people tend to anthropomorphise robots and exhibit social reactions, robot tears may elicit reactions similar to those of as human tears. Systematic experiments by Reeves and Nass. (1996) have shown that people behave socially and politely towards flattering inorganic objects, such as computers. This theory, which argues that people tend to assign human characteristics to media, is called media equation theory (Reeves and Nass, 1996). Furthermore, this phenomenon occurs unconsciously and automatically (Reeves and Nass, 1996), and has been confirmed in people’s reactions to robots (e.g., Rosenthal-von der Pütten et al., 2013; Suzuki et al., 2015). This phenomenon suggests that people tend to perceive inorganic objects anthropomorphically. Consequently, we predicted that people’s reactions to robot tears would be similar to their reactions to human tears. Specifically, we predicted that people would attribute a robot’s tears to feelings of sadness, thereby increasing the rating of sadness intensity. Furthermore, it was predicted that people would evaluate a robot shedding tears as warm and helpless, similar to a tearful person, and would feel more connected to the robot, thereby resulting in increased support intention.

## 3 Study 1

The participants viewed pictures of robots with and without tears and rated the intensity of the emotion experienced by the robot in the picture. In previous studies of human tears, the type of emotion for which ratings were asked varied from study to study, with some asking participants to respond to sadness only (Provine et al., 2009; Balsters et al., 2013), to negative emotions only (Ito et al., 2019), and to Ekman’s (1999) basic six emotions (Gračanin et al., 2021). It is also a customary and common practice in numerous facial expression recognition studies to measure Ekman’s (1999) six basic emotions (happiness, sadness, anger, disgust, fear, and surprise; e.g., Miwa et al., 2003; Ge et al., 2008; Kishi et al., 2012; Bennett and Šabanović, 2014). This study is the first to examine the impact of robot tears on emotional ratings. To determine the impact of more emotions, we asked the participants to rate the six basic emotions proposed by Ekman. (1999). Previous studies have shown that tears enhance sadness (e.g., Provine et al., 2009; Ito et al., 2019). Thus, we predicted that robots with tears would have higher ratings of sadness intensity than those without tears (Hypothesis 1). Conversely, the effect of tears on emotions other than sadness depends on the facial expressions before tears are applied. For example, applying tears to an angry facial expression results in a stronger evaluation of the anger emotion conveyed by the expression (Gračanin et al., 2021). However, because the robots used in this study were incapable of changing their facial expressions or clearly conveying an expression of a particular emotion, no explicit hypotheses were formulated for emotions other than sadness.

### 3.1 Materials and methods

#### 3.1.1 Participants

Fifty-two undergraduate students from a Japanese university participated in Study 1. Participants with one or more missing data were excluded. The final analysis included 50 participants (16 men, 34 women; mean age = 20.16 years, *SD* = 1.27). All participants were native Japanese speakers. Class participation points were awarded to participants of the experiment. Before conducting Study 1, all participants were provided with an overview of the study using Qualtrics, an online survey platform. After being provided an overview, participants were asked to select “agree” or “disagree” to participate in the study. Only participants who selected “agree” were included in the survey. The ideal sample size was calculated using G*Power (Faul et al., 2007) with a significance level of 5% and power of 0.80, considering the effect size (*d* = 0.86) of the meta-analysis conducted by Zickfeld et al. (2021). The power analysis results suggested 13 as the minimum sample size. The sample size for this study exceeded the minimum requirement.

#### 3.1.2 Visual stimulus

Four images of robots were used in this study. The robots were selected according to the following criteria set by Mathur and Reichling (2016). Furthermore, the signalling function of human tears has been shown to occur regardless of facial expression prior to the imposition of tears (Zeifman and Brown, 2011; Ito et al., 2019). Many robots are unable to change their facial expressions, and unlike humans, there are no standardised stimuli that represent each emotion. Thus, the facial expressions of the robots were not considered in their selection.

##### 3.1.2.1 Selection criteria

1. The entire face of the robot must be in the picture.
2. The robot’s face should be facing forward and both eyes should be in the picture.
3. The robot must be designed to interact socially with people.
4. The robot should have actually been produced.
5. The robot must physically move (not a sculpture or computer graphics).
6. Picture elucidation must be at least 100 d.p.i.

##### 3.1.2.2 Exclusion criteria

1. The robot represents a well-known character or celebrity.
2. The robot represents a specific gender.
3. The robot is sold as a toy.

**FIGURE 1:**
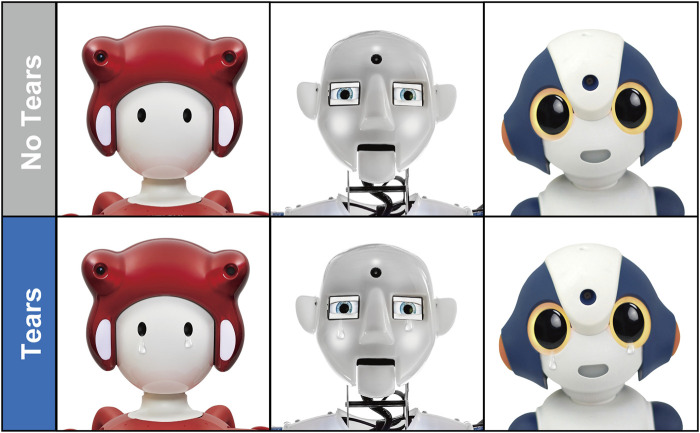
Visual stimuli used in the experiment. From the left, it is EMIEW, Robo Thespian, and Sota. The picture of RoBoHoN is not shown due to copyright issues.

Pictures from Hitachi Building Systems Co., Ltd.’s EMIEW, Sharp Corporation’s RoBoHoN, Engineered Arts Ltd.’s RoboThespian, and Vstone Co., Ltd.’s Sota, which met the above criteria and whose use was permitted, were used in the experiments. These pictures were obtained from company websites or provided directly by the companies. Pictures of the robots were cropped from the shoulders to the upper part of the body, and the background was white. Next, pictures of the four robots were digitally processed using Adobe Photoshop Cc 2021 to add tears (Figure 1). The size and position of the tears were standardised across robots. The final number of stimuli was eight: two pictures of each robot with and without tears. Furthermore, the suitability of tear processing was confirmed in a preliminary study and used in the main study. Details of the pilot studies are provided at https://osf.io/cgvt5/?view_only=39a3afb9ba724f669ea852e57a7afee1.

#### 3.1.3 Experimental design

The independent variable was the addition of tears (no tears: No Tears condition; with tears: Tears condition), which was a within-participants factor. The dependent variable was emotional intensity, which measured the intensity of the six emotions (sadness, anger, fear, surprise, disgust, and happiness) for each stimulus.

#### 3.1.4 Procedure

Participants were seated approximately 30 cm in front of a 26.5 cm × 47.0 cm stimulus presentation monitor and were surveyed using Qualtrics. A picture of the robot was presented at a size of 15.87 cm × 15.87 cm (600 px × 600 px), and participants were asked to rate on a scale of 101 (0: not at all strong to 100: very strong) how strongly they thought the robot in the picture was experiencing sadness, anger, fear, surprise, disgust, and happiness. The order in which the six emotions were presented was random for each trial. The number of stimuli used in this study was small, and each stimulus was presented three times to ensure measurement stability (Balsters et al., 2013; Gračanin et al., 2021). There were 24 trials in Study 1. In all 24 trials, stimuli were presented in random order using the Qualtrics randomisation function. The participants evaluated both versions of the same robot, with and without tears, and no time limit was placed on their responses. Study 1 was conducted after obtaining approval (KH-21088) from the Ethics Review Committee of the Faculty of Psychology at Doshisha University.

### 3.2 Result

**FIGURE 2:**
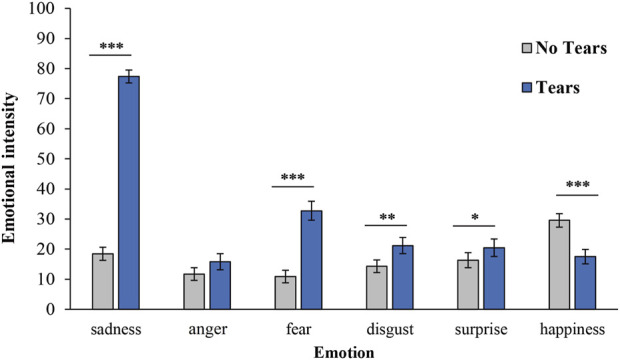
Mean of emotional intensity for the No Tears and Tears conditions. Asterisks indicate significant differences (*p < .05, **p < .01, ***p < .001). Error bars represent the standard error of the mean.

Data were analysed using IBM SPSS Statistics (v. 27). Each response for the four robots was combined for the No Tears and Tears conditions, and the mean of each condition was calculated (Figure 2). Cronbach’s alpha coefficient was calculated to check consistency between robots, which confirmed sufficient consistency (sadness: *α* = .90, anger: *α* = .81, fear: *α* = .83, disgust: *α* = .75, surprise: *α* = .78, happiness: *α* = .62). It should be noted that, in this study, we were interested in the average of the reactions to multiple robots, rather than the reaction to a unique robot. Therefore, the type of robot was not incorporated as a factor, but a *post hoc* analysis incorporating the imposition of tears and the type of robot as factors was conducted at the reviewer’s suggestion and is presented in the Supplementary Material.

As shown in Figure 2, the means of the six emotional intensities in the No Tears condition were below the median of 50. However, in the Tears condition, only sadness had a mean value higher than the median. A paired *t*-test was conducted to determine the difference in the intensity of each emotion depending on the addition of tears, using the means of the intensity of the emotions in the No Tears and Tears conditions. The results showed significant differences in emotions with the addition of tears except in anger [anger: *t* (49) = 1.91, *p* = .06, *d* = 0.27, 95% CI (−0.01, 0.55), sadness: *t* (49) = 24.18, *p* < .001, *d* = 3.42, 95% CI (2.69, 4.15), fear: *t* (49) = 8.96, *p* < .001, *d* = 1.27, 95% CI (0.89, 1.64), surprise: *t* (49) = 2.10, *p* = .04, *d* = 0.30, 95% CI (0.01, 0.58), disgust: *t* (49) = 3.10, *p* = .003, *d* = 0.44, 95% CI (0.15, 0.73), happiness: *t* (49) = −5.14, *p* < .001, *d* = −0.73, 95% CI (−1.04, −0.41)]. The emotional intensity of sadness, fear, surprise, and disgust were significantly higher in the Tears condition than in the No Tears condition, whereas the emotional intensity of happiness was significantly lower in the Tears condition than in the No Tears condition.

### 3.3 Discussion

Study 1 investigated whether robot tears also serve as emotional signals for conveying sadness. We hypothesised that robots that shed tears would receive higher ratings for intensity of sadness than robots that did not shed tears (Hypothesis 1). According to the results, the Tears condition had significantly higher ratings for the intensity of sadness compared to the No Tears condition, thereby supporting the hypothesis. Additionally, the Tears condition showed higher ratings of emotional intensity than the No Tears condition for the emotions of fear, surprise, and disgust.

Furthermore, the results of Study 1 indicate that robots with tears have the emotional signalling function of conveying sadness. Compared with the No Tears condition, the Tears condition showed a significantly higher sadness intensity rating of 77.36, which was above the median. The intensity of happiness was lower in the Tears condition than in the No Tears condition. These results are consistent with those of previous studies showing that tears enhance sadness (Provine et al., 2009; Zeifman and Brown, 2011; Balsters et al., 2013; Ito et al., 2019; Gračanin et al., 2021). Conversely, tears may enhance the intensity of emotions other than sadness (fear, surprise, and disgust). Shedding tears is an emotionally expressive behaviour associated with emotions other than sadness and occurs as a result of strong emotional arousal (Vingerhoets et al., 2001). Consequently, the addition of tears may increase the intensity of emotions other than sadness in the present study. However, any increase in the emotional intensity of emotions other than sadness due to the addition of tears was below the median, and the effect sizes were smaller for the other emotions (fear, *d* = 1.27; surprise, *d* = 0.30; disgust, *d* = 0.44) than for sadness (*d* = 3.42). In other words, it can be inferred that the enhancing effect of tears on emotions other than sadness is secondary. Thus, this study demonstrated that tears enhanced sadness the most, that is, also had the emotional signalling function of conveying sadness.

The reason robot tears augment sadness may be influenced by the characteristics of people’s anthropomorphic perceptions of media, such as computers and television. Anthropomorphism refers to the general tendency of people to attribute human-specific characteristics (Epley et al., 2007; Waytz et al., 2010), including human-like mental abilities, to non-human objects. People tend to anthropomorphise robots (e.g., Tanaka et al., 2007; Rosenthal-von der Pütten et al., 2013). Human tears enhance various facial expressions (e.g., Provine et al., 2009; Zeifman and Brown, 2011; Balsters et al., 2013; Ito et al., 2019; Gračanin et al., 2021). Therefore, even if an inorganic robot is shedding tears, people perceive it in the same way as a tearful individual and interpret the robot as experiencing sadness. This suggests that the robot’s tears increased the emotional intensity of sadness in the present study.

## 4 Study 2

Study 1 revealed that tears enhance the rated intensity of sadness in robots. Study 2 aimed to investigate whether robot tears serve as a social signal to elicit support intentions using different scenarios. We also examined whether the effect of tears on eliciting support intentions was mediated by perceptions of warmth, helplessness, and connectedness, as in humans. Because many previous studies have shown that tears have a strong effect on eliciting support intentions (e.g., Vingerhoets et al., 2016; Stadel et al., 2019; Zickfeld et al., 2021), it was hypothesised that support intentions would be higher for robots with tears than for robots without tears (Hypothesis 2). Furthermore, the tear-eliciting support intention effect is mediated by increased perceptions of warmth, helplessness, and connectedness of the tearful individual (Vingerhoets et al., 2016; Zickfeld et al., 2021). We also hypothesised that the effect of tears on eliciting support intentions would be mediated by the perceptions of warmth, helplessness, and connectedness (Hypothesis 3).

### 4.1 Materials and methods

#### 4.1.1 Participants

Fifty-six undergraduate students (22 men and 34 women, mean age = 20.18 years, *SD* = 1.19 years) from a Japanese university agreed to participate in Study 2. All participants were native Japanese speakers. Class participation points were awarded to participants of the experiment. Before conducting Study 2, all participants were provided with an overview of the study using Qualtrics, an online survey platform. After this, participants were asked to select “agree” or “disagree” to participate in the study. Only participants who selected “agree” were included in the survey. The ideal sample size was calculated using G*Power (Faul et al., 2007) with a significance level of 5% and a power of 0.80, considering the effect size (*d* = 0.56) of the meta-analysis conducted by Zickfeld et al. (2021). The power analysis results suggested 28 as the minimum sample size. The sample size for this study exceeded the minimum requirement.

#### 4.1.2 Visual stimulus

The same pictures of the robots used in Study 1 were utilised.

#### 4.1.3 Scenarios

We created two scenarios to be presented with the robot’s picture, one on the theme of “death” and the other on the theme of “farewell,” which are the antecedents that typically precede crying behaviour (Vingerhoets, 2013). The first scenario was “It has just been decided in front of the robot that this robot will be dismantled tomorrow.” The second was “The rental period for this robot ends today, and this robot is just now leaving the family it is with.”

#### 4.1.4 Measures

The present study used the same questionnaire items as those used by Zickfeld et al. (2021) to measure support intentions and perceptions of warmth, helplessness, and connectedness.

##### 4.1.4.1 Support intentions

To measure the intention to support the robot in the picture, participants were asked to respond to the following three items using a 7-point scale (0: not at all to 6: very much so): “I would be there if this robot needed me,” “I would express how much I accept this robot,” and “I would offer support to this robot.”

##### 4.1.4.2 Perceived warmth

To measure the perceived warmth towards the robot in the picture, participants were asked to indicate the extent to which the two items—“warm” and “friendly”—applied to the robot in the picture using a 7-point scale (from 0: not at all to 6: very much so).

##### 4.1.4.3 Perceived helplessness

To measure the perceived helplessness of the robot in the picture, participants were asked to respond to the following three items using a 7-point scale (0: not at all to 6: very much so): “How helpless does this robot appear to you?”, “How overwhelmed does this robot appear to you?”, and “How sad does this robot appear to you?”

##### 4.1.4.4 Perceived connectedness

To measure the perceived connectedness to the robot in the picture, participants were asked to rate their perception of connectedness to the robot in the picture on a 7-point IOS (Inclusion of others in the self) scale (Aron et al., 1992). The IOS scale consists of seven figures, ranging from two separate circles, the self and the other, to two almost overlapping circles.

#### 4.1.5 Experimental design

The independent variable was the addition of tears (no tears: No Tears condition, with tears: Tears condition), which was a within-participant factor. The dependent variables were support intention, perceived warmth, perceived helplessness, and perceived connectedness.

#### 4.1.6 Procedure

Participants were seated approximately 30 cm in front of a 26.5 cm × 47.0 cm stimulus presentation monitor and were surveyed using Qualtrics. A picture of the robot was presented at a size of 15.87 cm × 15.87 cm (600 px × 600 px) along with the scenario, and participants were asked to answer the questions. The participants performed 16 trials (four robots × with/without tears × two scenarios). The order of stimulus presentation was random using the Qualtrics randomisation function for all 16 trials, and no time limit was placed on the responses. Participants evaluated both versions of the same robot, with and without tears. Study 2 had more questions than Study 1 and did not use the technique of asking participants to rate the same visual stimulus three times in order to reduce their burden. However, despite the difference in the type of scenario, the fact that the evaluation was asked twice for one visual stimulus suggests that the stability of the measurement was ensured. Study 2 was conducted after obtaining approval (KH-21088) from the Ethics Review Committee of the Faculty of Psychology at Doshisha University.

### 4.2 Result

Data were analysed using IBM SPSS Statistics (v. 27). The mean of the three items on support intentions was used as the support intention score (*α* = .92). Similarly, means of the two items for perceived warmth and three items for perceived helplessness were treated as their respective scale scores (warmth: *r* = .82; helplessness: *α* = .74).

#### 4.2.1 Effect of tears

**FIGURE 3:**
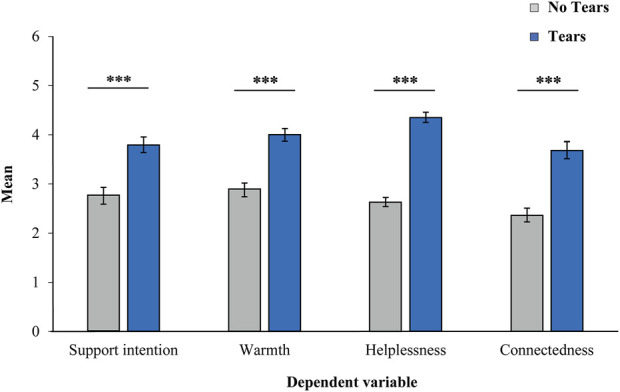
Mean of each dependent variable for the No Tears and Tears conditions. Asterisks indicate significant differences (***p < .001). Error bars represent the standard error of the mean.

The responses to the four robots and the two scenarios were grouped according to the No Tears and Tears conditions, and the average was calculated for each condition. As shown in Figure 3, the means in the Tears condition were higher than those in the No Tears condition for all the dependent variables. A paired *t*-test was conducted to examine whether the addition of tears produced differences in perceived support intention, warmth, helplessness, and connectedness for each dependent variable, using the means of the No Tears and Tears conditions. Results showed significant differences in all dependent variables, with higher scores in the Tears condition as compared to the No Tears condition [Support intention: *t* (55) = 7.89, *p* < .001, *d* = 1.05, 95% CI (0.72, 1.38); Warmth: *t* (55) = 9.31 *p* < .001, *d* = 1.24, 95% CI (0.89, 1.59); Helplessness: *t* (55) = 15.48, *p* < .001, *d* = 2.07, 95% CI (1.60, 2.53); Connectedness: *t* (55) = 10.98, *p* < .001, *d* = 1.47, 95% CI (1.09, 1.84)].

#### 4.2.2 Mediation analysis

**FIGURE 4:**
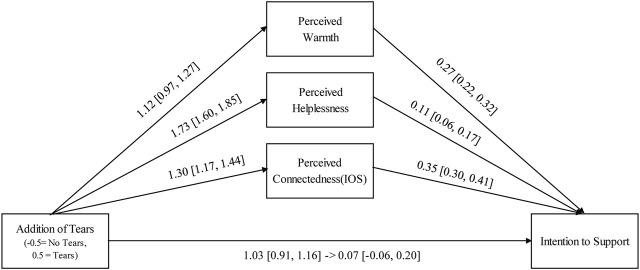
Mediation model summarising the direct and indirect effects of the occurrence of tears on the intention to support. Coefficients represent unstandardised estimates. Estimate in parentheses represents 95% confidence intervals.

A mediation analysis was performed using R (ver. 4.2.1) to determine whether the relationship between the addition of tears and support intentions was mediated by perceptions of warmth, helplessness and connectedness (Hypothesis 3). The mediation analysis was performed with the addition of tears as the independent variable, support intention as the dependent variable, and perceived warmth, helplessness, and connectedness as the mediating variables (Figure 4). As this experimental study utilised a repeated measures design, a multi-model mediation analysis was conducted with participants included as random intercepts. Monte Carlo simulations were employed to construct 95% confidence intervals for the indirect effect of the addition of tears on support intentions via the mediating variables (Falk and Biesanz, 2016). The results showed that the 95% confidence intervals for indirect effects on all mediating variables did not include zero [warmth: *B* = 0.31, 95% CI (0.24, 0.38); helplessness: *B* = 0.20, 95% CI (0.10, 0.29); and connectedness: *B* = 0.47, 95% CI (0.38, 0.55)].

### 4.3 Discussion

Study 2 examined whether robot tears served as social signals that elicited support intentions. We also examined whether the effect of tears on support intention was mediated by perceived warmth, helplessness, and connectedness. It was hypothesised that the Tears condition would result in higher support intentions than the No Tears condition (Hypothesis 2). The results showed that support intentions were significantly higher in the Tears condition than in the No Tears condition, thereby supporting this hypothesis. Furthermore, we hypothesised that the effect of tears on eliciting support intentions was mediated by perceived warmth, helplessness, and connectedness (Hypothesis 3). The results of this study replicated previous findings: tears not only made the expresser appear helpless, but also made others feel warmer and more connected to them (Stadel et al., 2019; Zickfeld et al., 2021). Furthermore, the effect of tears on eliciting support intentions was mediated by perceived warmth, helplessness, and connectedness, thereby supporting Hypothesis 3. Thus, the results suggest that, even in robots, tears can function as social signals that elicit support intention.

The results of this study demonstrate for the first time that robot tears have a social signalling function to elicit support intentions, thereby supporting the media equation theory proposed by Reeves and Nass. (1996). The media equation theory states that people behave socially towards inorganic objects, such as media, and tend to perceive them as anthropomorphic. In a study conducted by Reeves and Nass. (1996), the social behaviours that occur in person-to-person relationships were replicated in person-to-media relationships. Therefore, the social behaviour that occurs in person-to-person relationships wherein support intentions are elicited towards a tearful individual was also demonstrated in person-to-robot relationships in this study.

Additionally, the present study showed that the process by which tears cause an increase in support intention may be similar to that in humans. In the present study, tearful robots received higher ratings for perceived warmth, helplessness, and connectedness than non-tearful robots. Furthermore, these three factors mediated the effect of tears on increased support intentions, thereby replicating the results of previous studies conducted with human pictures (Stadel et al., 2019; Zickfeld et al., 2021). In addition to the signal effect of eliciting support intentions, this study showed that the process of eliciting support intentions by the robot’s tears was similar to that of humans, suggesting that people may perceive the robot as similar to a person.

## 5 General discussion

The present study aimed to investigate whether tears shed by robots have an emotional signalling function for conveying sadness and a social signalling function for eliciting support intentions, using methods similar to those of previous studies conducted on human tears. Study 1 examined whether robot tears served as an emotional signal to convey sadness, and demonstrated that robots with tears conveyed enhanced sadness. Study 2 examined whether robot tears serve as a social signal to elicit support intentions and found that robot tears also elicit support intentions. Moreover, the process by which robot tears elicit support intentions was found to be similar to that of humans.

The results of the present study suggest that tears may have facilitated the anthropomorphisation of the robot. The results of the two experiments conducted in the present study replicated those of previous studies using human pictures. This demonstrated, for the first time, that tears in robots may function as emotional and social signals in the same manner as in humans. As inorganic objects, robots have no emotions or will and do not shed tears spontaneously. However, because people tend to anthropomorphise inorganic robots (Reeves and Nass, 1996), it is presumed that they perceive emotions and sociality in the tears of the robots. In addition to this flexibility in human cognition, it is possible that the object itself, the tears, may have been a catalyst for people to find emotional and social qualities in the robot and may have promoted anthropomorphising and perception of the robot with human-like characteristics. Riek et al. (2009) examined whether people showed empathy towards robots by displaying videos of robots being abused. The results showed that there were differences in the degree of empathy depending on the appearance of the abused robot, with higher empathy shown for a human-like robot (android) than for a robot with a mechanical appearance (cleaning robot Roomba). Thus, the personhood of a robot influences people’s social responses to it. Moreover, the perception of a robot’s personhood has been argued to increase acceptance, liking, familiarity and trust towards the robot and facilitate social interactions with people (Eyssel et al., 2010; Fink, 2012). Because shedding tears is a unique human expression of emotions (Vingerhoets, 2013), it can be inferred that tears are symbolic elements of personhood. Therefore, the addition of tears to a robot, which is an inorganic object, may have promoted people’s tendency to perceive inorganic objects as anthropomorphic and the robot as an infinitely human-like entity.

Furthermore, shedding tears is a meaningful way for robots to express their emotions. The results of this study suggest that robot tears increase the intensity of sadness and that shedding tears can function as an emotionally expressive behaviour of sadness in robots. In addition to sadness, tears are accompanied by diverse emotions such as happiness and anger (Vingerhoets, 2013). Although Study 1 did not provide contextual information, it may be possible to express emotions other than sadness through tears, depending on the context in which the tears are shed. Consequently, equipping robots with tear-shedding behaviour is expected to broaden their range of emotional expressions and make human-robot interactions more sophisticated and richer. Moreover, the perception of sad facial expressions differs between young and older adults, with older adults being less sensitive to the perception of sad facial expressions compared to young adults (Phillips and Allen, 2004; MacPherson et al., 2006). However, no differences were found between older and young adults in their assessment of grief in response to the emotional expression of shedding tears, indicating that tears are a universal emotional signal that conveys grief across all ages (Grainger et al., 2019). Therefore, the provision of a tear function as a way of expressing sadness in robots may be beneficial, as it facilitates the transmission of sadness regardless of the age of the person interacting with the robot.

This study demonstrates for the first time that the social functioning of emotions can occur when robots shed tears. It has been shown that when robots express emotions through facial expressions and gestures, they exhibit the same social functions as humans (Melo et al., 2023). The present study supports the results of previous studies and shows that this applies to the previously untested emotional expression of shedding tears, thereby contributing to new knowledge in this area. Specifically, Study 2 showed that robot tears elicited support intentions and influenced human behaviours. The behaviour of shedding tears attracts the attention of others and elicits approach responses (Hendriks et al., 2008; Vingerhoets, 2013). Moreover, it can be applied for the treatment of autism through human-robot interactions (e.g., Scassellati et al., 2012; Taheri et al., 2018) and as “robot being cared for” to fulfil the self-esteem and self-affirmation of older adults (Kanoh, 2014). In other words, equipping robots with the ability to shed tears may contribute to further developments in fields where robots are actively utilised.

Finally, the limitations and future perspectives of this study are discussed. First, the baseline of stimuli used in this study was not uniform. For example, some robots missed certain facial parts (mouth and nose) or appeared to smile. In humans, tears have been shown to enhance the evaluation of sadness and support intentions, regardless of facial expressions prior to the imposition of tears (e.g., Provine et al., 2009; Zeifman and Brown, 2011; Ito et al., 2019). However, the magnitude of the effect of tears varies across facial expressions (e.g., Ito et al., 2019). Therefore, future work is needed to control the facial expressions and parts of the robot and examine the effect of tears. In addition, the number of robot types used in the experiments was small. Although four different types of robots were used in this study, many different types of communicative robots have been developed with varying appearances, ranging from those modelled on animals, such as dogs and seals, to those that look more like humans (Weiss et al., 2009; Zecca et al., 2009; Eyssel et al., 2010; Pandey and Gelin, 2018). Consequently, it is unclear whether the results presented in this study can be generalised to all robots. Second, contextual information was limited. Specifically, Study 1 did not include any contextual information. This is a necessary procedure for identifying the emotions signified by tears. Contextual information is a very important factor in determining the emotion indicated by the emotional expression, especially tears, which can be associated with a range of emotions (Vingerhoets, 2013; Zickfeld et al., 2020). Future studies should set contextual information and determine whether a robot’s tears can also intensify the evaluation of other emotional expressions such as joy, emotion, and anger. Third, the present study dealt only with subjective assessments. Our aim was to identify the signalling function of robot tears using an approach similar to that of previous studies conducted on human tears. Thus, we asked the participants to respond to the support intention of a subjective evaluation of the pictures of the robot. This method is important because it provides the first clues to actual human reactions to the robot. Conversely, previous studies examining the social function of emotions have shown that compared to non-expressed emotions, robots made concessions in negotiation games to opponents who showed emotional expressions (Sinaceur et al., 2015), donated more money (Takagi and Terada, 2021), and made suggestions with high amounts of money in ultimatum games (Terada and Takeuchi, 2017), showing that emotional expressions can influence the behaviour of others. In the present study, support intentions increased for robots that shed tears. However, it is not clear whether this leads to actual support behaviour. Future hypothesis testing using behavioural indicators is required.

## 6 Conclusion

The tears in robots may have signalling effects similar to those in humans. Robot tears have enhanced the rated intensity of sadness. They also indicated warmth and helplessness. The observers felt a sense of closeness to the tear-shedding robot, which led to support intentions. The results suggest that robot tears, like human tears, have both an emotional signalling function for conveying sadness and a social signalling effect that elicits support intentions. This is the first study to demonstrate the previously unidentified emotional and social signalling functions of robot tears and to determine the potential for new tear-specific interactions between humans and robots.

## Data Availability

The datasets presented in this study can be found in online repositories. The URL to access repository can be found below: https://osf.io/cgvt5/?view_only=39a3afb9ba724f669ea852e57a7afee1.
