# Supplementary material for: Robots with tears can convey enhanced sadness and elicit support intentions
Source: Front Robot AI. 2023 Jun 1;10:1121624. doi: 10.3389/frobt.2023.1121624 (PMC10267379; doi:10.3389/frobt.2023.1121624)
Supplement: Supplementary file 1 [file DataSheet1.DOCX]

Supplementary Material

Robots with Tears can Convey Enhanced Sadness and Elicit Support Intentions

Akiko Yasuhara*, Takuma Takehara

*** Correspondence:** Akiko Yasuhara: a.yasuhara.do@gmail.com

# Post-hoc analysis

This section reports the results of an exploratory test of the effects of robot type (Study 1). A two-way repeated measures ANOVA was conducted to test whether there were differences in the mean value of each emotional intensity between the addition of tears and robot type, with the addition of tears and robot type as independent variables and the mean value of each emotional intensity as the dependent variable. We analysed the data using the anovakun package in R version 4.2.1. If the results of the sphericity test were significant, the Greenhouse-Keizer method corrected degrees of freedom were used. Multiple comparisons were performed using Shaffer's modified sequentially rejective Bonferroni procedure.

## Robot type

Four types of robots were used in this study (Figure 1).

They are denoted below as RoBoHoN = b1, Robothesipian = b2, EMIEW = b3, and Sota = b4.


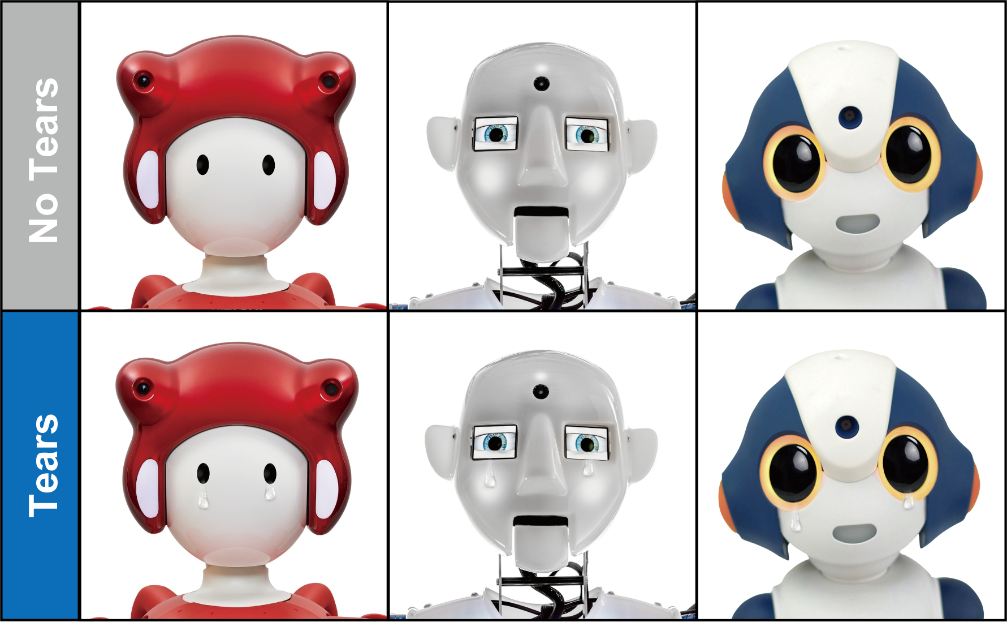


**Figure 1. Visual stimuli used in the experiment.** From the left, it is EMIEW, Robo Thespian, and Sota. The photo of RoBoHoN is not shown owing to copyright issues.

## Sadness

The mean values for each condition and robot type are shown in Figure 2. We conducted a two-factor repeated measures ANOVA, and the results showed a significant difference in the main effects of the addition of tears (*F*(1, 49) = 584.75, *p*<.001, *η*^2^ = 0.60), main effects of robot type (*F*(2.20,107.92) = 54.60, *p*<.001, *η*^2^ = 0.08), and interaction effects (*F*(2.39, 117.31) = 13.77, *p*<.001, *η*^2^ = 0.02). The results of the simple main effect tests are presented in Table 1. The simple main effect of the addition of tears for all robot types was significant. Thus, the mean value of the Tears condition was significantly higher than that of the No tears condition. The robot type simple main effects in the No tears and Tears conditions were also significant; therefore, tests of multiple comparisons were conducted (Table 2).

**Figure 2. Mean of emotional intensity of sadness for each condition and type of robot.** Error bars represent the standard error of the mean.

**Table 1. Results of the simple main effect test of sadness**. A= The addition of tears factor, a1=No tears condition, a2=Tears condition, B= Robot type factor.

|  | **SS** | **df** | **MS** | ***F*** | ***p*** | **η^2^** |
| --- | --- | --- | --- | --- | --- | --- |
| **A at b1** | 93228.24 | 1 | 93228.24 | 289.09 | <.001 | 0.68 |
| **Error (A at b1)** | 15801.74 | 49 | 322.48 |  |  |  |
| **A at b2** | 44253.01 | 1 | 44253.01 | 106.53 | <.001 | 0.46 |
| **Error (A at b2)** | 20355.21 | 49 | 415.41 |  |  |  |
| **A at b3** | 128976.51 | 1 | 128976.5 | 435.99 | <.001 | 0.79 |
| **Error (A at b3)** | 14495.41 | 49 | 295.82 |  |  |  |
| **A at b4** | 92010.3 | 1 | 92010.3 | 234.98 | <.001 | 0.67 |
| **Error (A at b4)** | 19186.59 | 49 | 391.56 |  |  |  |
| **B at a1** | 44929.16 | 1.86 | 24142.36 | 58.25 | <.001 | 0.35 |
| **Error (B at a1)** | 37795.86 | 91.19 | 414.48 |  |  |  |
| **B at a2** | 13883.72 | 2.21 | 626868 | 14.96 | <.001 | 0.13 |
| **Error (B at a2)** | 45466.42 | 108.52 | 418.95 |  |  |  |

**Table 2. Results of multiple comparisons of sadness.** Asterisks indicate significant differences (****p* <.001).

| **Robot type in the No tears condition** | | | | | | |
| --- | --- | --- | --- | --- | --- | --- |
| **Pair** | **diff** | ***t*** | ***df*** | ***p*** |  |  |
| **b1-b2** | -35.41 | 8.74 | 49 | <.001 |  | **b1 < b2***** |
| **b1-b3** | -4.53 | 2.2 | 49 | 0.10 |  | **b1 = b3** |
| **b1-b4** | 1.11 | 0.59 | 49 | 0.56 |  | **b1 = b4** |
| **b2-b3** | 30.87 | 8.16 | 49 | <.001 |  | **b2 > b3***** |
| **b2-b4** | 36.52 | 8.98 | 49 | <.001 |  | **b2 > b4***** |
| **b3-b4** | 5.65 | 2.2 | 49 | 0.10 |  | **b3 = b4** |
|  | | | | | | |
| **Robot type in the Tears condition** | | | | | | |
| **Pair** | **diff** | ***t*** | ***df*** | ***p*** |  |  |
| **b1-b2** | -16.41 | 4.42 | 49 | <.001 |  | **b1 < b2***** |
| **b1-b3** | -15.29 | 4.76 | 49 | <.001 |  | **b1 < b3***** |
| **b1-b4** | 1.51 | 0.49 | 49 | 1.00 |  | **b1 = b4** |
| **b2-b3** | 1.12 | 0.45 | 49 | 1.00 |  | **b2 = b3** |
| **b2-b4** | 17.93 | 4.29 | 49 | <.001 |  | **b2 > b4***** |
| **b3-b4** | 16.81 | 4.08 | 49 | <.001 |  | **b3 > b4***** |

## Anger

The mean values for each condition and robot type are shown in Figure 3. We conducted a two-factor repeated measures ANOVA, and the results showed no significant difference in the main effects of the addition of tears (*F*(1, 49) = 3.66, *p* = .06, *η*^2^ = 0.01), but showed a significant main effect of robot type (*F*(2.35,115.31) = 24.52, *p*<.001, *η*^2^ = 0.08) and interaction effects (*F*(2.16, 106.08) = 8.53, *p*<.001, *η*^2^ = 0.01). The results of the simple main effect tests are presented in Table 3. The simple main effect of the addition of tears on only b3 and b4 were significant. Thus, in b3 and b4, the mean of the Tears condition was significantly higher than that of the No tears condition. The effects of the robot type simple main effects in the No tears and Tears conditions were also significant; therefore, tests of multiple comparisons were conducted (Table 4).

**Figure 3. Mean of emotional intensity of anger for each condition and type of robot.** Error bars represent the standard error of the mean.

**Table 3. Results of the simple main effect test of anger**. A= The addition of tears factor, a1=No tears condition, a2=Tears condition, B= Robot type factor.

|  | **SS** | **df** | **MS** | ***F*** | ***p*** | **η^2^** |
| --- | --- | --- | --- | --- | --- | --- |
| **A at b1** | 456.59 | 1 | 456.59 | 3.09 | 0.09 | 0.01 |
| **Error (A at b1)** | 7247.74 | 49 | 147.91 |  |  |  |
| **A at b2** | 2.12.17 | 1 | 212.17 | 0.94 | 0.34 | 0.004 |
| **Error (A at b2)** | 11063.51 | 49 | 225.79 |  |  |  |
| **A at b3** | 1137.98 | 1 | 1137.98 | 6.40 | 0.01 | 0.03 |
| **Error (A at b3)** | 14495.41 | 49 | 295.82 |  |  |  |
| **A at b4** | 1711.06 | 1 | 1711.06 | 13.97 | <.001 | 0.08 |
| **Error (A at b4)** | 6002.40 | 49 | 122.50 |  |  |  |
| **B at a1** | 11577.58 | 1.98 | 5861.82 | 25.21 | <.001 | 0.15 |
| **Error (B at a1)** | 22502.54 | 96.78 | 232.51 |  |  |  |
| **B at a2** | 2642.18 | 3 | 880.73 | 10.11 | <.001 | 0.03 |
| **Error (B at a2)** | 12809.85 | 147 | 87.14 |  |  |  |

**Table 4. Results of multiple comparisons of anger.** Asterisks indicate significant differences (**p* <.05, ****p* <.001).

| **Robot type in the No tears condition** | | | | | | |
| --- | --- | --- | --- | --- | --- | --- |
| **Pair** | **diff** | ***t*** | ***df*** | ***p*** |  |  |
| **b1-b2** | -16.09 | 5.76 | 49 | <.001 |  | **b1 < b2***** |
| **b1-b3** | -1.16 | 0.80 | 49 | 0.43 |  | **b1 = b3** |
| **b1-b4** | 3.94 | 2.30 | 49 | 0.06 |  | **b1 = b4** |
| **b2-b3** | 14.93 | 4.73 | 49 | <.001 |  | **b2 > b3***** |
| **b2-b4** | 20.03 | 6.52 | 49 | <.001 |  | **b2 > b4***** |
| **b3-b4** | 5.10 | 2.41 | 49 | 0.02 |  | **b3 > b4*** |
|  | | | | | | |
| **Robot type in the Tears condition** | | | | | | |
| **Pair** | **diff** | ***t*** | ***df*** | ***p*** |  |  |
| **b1-b2** | -8.91 | 4.08 | 49 | <.001 |  | **b1 < b2***** |
| **b1-b3** | -3.63 | 2.06 | 49 | 0.13 |  | **b1 = b3** |
| **b1-b4** | -0.06 | 0.04 | 49 | 0.97 |  | **b1 = b4** |
| **b2-b3** | 5.27 | 2.68 | 49 | 0.03 |  | **b2 > b3*** |
| **b2-b4** | 8.85 | 4.56 | 49 | <.001 |  | **b2 > b4***** |
| **b3-b4** | 3.57 | 2.03 | 49 | 0.13 |  | **b3 = b4** |

## Fear

The mean values for each condition and robot type are shown in Figure 4. We conducted a two-factor repeated measures ANOVA, and the results showed a significant difference in the main effects of the addition of tears (*F*(1, 49) = 3.66, *p* = .06, *η*^2^ = 0.01) and the main effects of robot type (*F*(2.35,115.31) = 24.52, *p*<.001, *η*^2^ = 0.08), but showed no significant interaction effects (*F*(2.16, 106.08) = 8.53, *p*<.001, *η*^2^ = 0.01). Thus, the mean value of the Tears condition was significantly higher than that of the No tears condition. The results of multiple comparisons of the main effects of robot type are presented in Table 5.

**Figure 4. Mean of emotional intensity of fear for each condition and type of robot.** Error bars represent the standard error of the mean.

**Table 5. Results of multiple comparisons of fear.** Asterisks indicate significant differences (**p* <.05, ***p* <.01, ****p* <.001).

| **Pair** | **diff** | ***t*** | ***df*** | ***p*** |  |  |
| --- | --- | --- | --- | --- | --- | --- |
| **b1-b2** | -14.02 | 6.12 | 49 | <.001 |  | **b1 < b2***** |
| **b1-b3** | -5.70 | 3.15 | 49 | 0.008 |  | **b1 < b3**** |
| **b1-b4** | -0.69 | 0.40 | 49 | 0.69 |  | **b1 = b4** |
| **b2-b3** | 8.32 | 3.65 | 49 | 0.002 |  | **b2 > b3**** |
| **b2-b4** | 13.33 | 5.68 | 49 | <.001 |  | **b2 > b4***** |
| **b3-b4** | 5.01 | 2.41 | 49 | 0.08 |  | **b3 = b4** |

## Disgust

The mean values for each condition and robot type are shown in Figure 2. We conducted a two-factor repeated measures ANOVA, and the result showed a significant difference in main effects of addition of tears (*F*(1, 49) = 9.63, *p* = .003, *η*^2^ = 0.02), main effects of robot type (*F*(1.62, 79.48) = 34.96, *p*<.001, *η*^2^ = 0.15), and the interaction effects (*F*(2.32, 113.92) = 12.16, *p*<.001, *η*^2^ = 0.02). The results of the simple main effect tests are presented in Table 6. The simple main effect of the addition of tears for all robot types was significant. Thus, in b1, b3, and b4, the mean of the Tears condition was significantly higher than that of the No tears condition. The robot type simple main effects in the No tears and Tears conditions were also significant; therefore, tests of multiple comparisons were conducted (Table 7).

**Figure 5. Mean of emotional intensity of disgust for each condition and type of robot.** Error bars represent the standard error of the mean.

**Table 6. Results of the simple main effect test of disgust**. A= The addition of tears factor, a1=No tears condition, a2=Tears condition, B= Robot type factor.

|  | **SS** | **df** | **MS** | ***F*** | ***p*** | **η^2^** |
| --- | --- | --- | --- | --- | --- | --- |
| **A at b1** | 1775.27 | 1 | 1775.27 | 10.88 | 0.002 | 0.05 |
| **Error (A at b1)** | 7998.85 | 49 | 163.24 |  |  |  |
| **A at b2** | 385.42 | 1 | 385.42 | 1.22 | 0.28 | 0.005 |
| **Error (A at b2)** | 15503.67 | 49 | 316.40 |  |  |  |
| **A at b3** | 3631.87 | 1 | 3631.87 | 16.89 | <.001 | 0.07 |
| **Error (A at b3)** | 10538.62 | 49 | 215.07 |  |  |  |
| **A at b4** | 3025.11 | 1 | 3525.11 | 22.70 | <.001 | 0.12 |
| **Error (A at b4)** | 6530.83 | 49 | 133.28 |  |  |  |
| **B at a1** | 28815.13 | 1.74 | 16572 | 42.23 | <.001 | 0.27 |
| **Error (B at a1)** | 33437.15 | 85.2 | 392.45 |  |  |  |
| **B at a2** | 7429.57 | 1.98 | 3757.79 | 12.97 | <.001 | 0.07 |
| **Error (B at a2)** | 28075.59 | 96.88 | 289.80 |  |  |  |

**Table 7. Results of multiple comparisons of disgust.** Asterisks indicate significant differences (**p* <.05, ****p* <.001).

| **Robot type in the No tears condition** | | | | | | |
| --- | --- | --- | --- | --- | --- | --- |
| **Pair** | **diff** | ***t*** | ***df*** | ***p*** |  |  |
| **b1-b2** | -26.77 | 7.06 | 49 | <.001 |  | **b1 < b2***** |
| **b1-b3** | -1.67 | 1.12 | 49 | 0.27 |  | **b1 = b3** |
| **b1-b4** | 3.51 | 1.91 | 49 | 0.12 |  | **b1 = b4** |
| **b2-b3** | 25.10 | 6.83 | 49 | <.001 |  | **b2 > b3***** |
| **b2-b4** | 30.28 | 7.73 | 49 | <.001 |  | **b2 > b4***** |
| **b3-b4** | 5.18 | 2.16 | 49 | 0.11 |  | **b3 = b4** |
|  | | | | | | |
| **Robot type in the Tears condition** | | | | | | |
| **Pair** | **diff** | ***t*** | ***df*** | ***p*** |  |  |
| **b1-b2** | -14.41 | 4.39 | 49 | <.001 |  | **b1 < b2***** |
| **b1-b3** | -5.29 | 2.40 | 49 | 0.04 |  | **b1 < b3*** |
| **b1-b4** | 0.94 | 0.60 | 49 | 0.55 |  | **b1 = b4** |
| **b2-b3** | 9.12 | 2.64 | 49 | 0.03 |  | **b2 > b3*** |
| **b2-b4** | 15.35 | 4.52 | 49 | <.001 |  | **b2 > b4***** |
| **b3-b4** | 6.23 | 3.02 | 49 | 0.01 |  | **b3 > b4*** |

## Surprise

The mean values for each condition and robot type are shown in Figure 5. We conducted a two-factor repeated measures ANOVA, and the results showed a significant difference in the main effects of the addition of tears (*F*(1, 49) = 4.43, *p* = .04, *η*^2^ = 0.008) and main effects of robot type (*F*(2.48,121.37) = 5.74, *p =* .002, *η*^2^ = 0.03), but showed no significant interaction effects (*F*(2.43, 118.91) = 0.58, *p* = 0.60, *η*^2^ = 0.001). Thus, the mean value of the Tears condition was significantly higher than that of the No tears condition. The results of multiple comparisons of the main effects of the robot type are presented in Table 8.

**Figure 6. Mean of emotional intensity of surprise for each condition and type of robot.** Error bars represent the standard error of the mean.

**Table 8. Results of multiple comparisons of surprise.** Asterisks indicate significant differences (**p* <.05, ***p* <.01).

| **Pair** | **diff** | ***t*** | ***df*** | ***p*** |  |  |
| --- | --- | --- | --- | --- | --- | --- |
| **b1-b2** | -3.51 | 1.33 | 49 | 0.56 |  | **b1 = b2** |
| **b1-b3** | 3.22 | 1.33 | 49 | 0.56 |  | **b1 = b3** |
| **b1-b4** | -7.19 | 3.22 | 49 | 0.01 |  | **b1 < b4**** |
| **b2-b3** | 6.73 | 2.93 | 49 | 0.02 |  | **b2 > b3*** |
| **b2-b4** | -3.67 | 1.18 | 49 | 0.56 |  | **b2 = b4** |
| **b3-b4** | -10.41 | 3.43 | 49 | 0.01 |  | **b3 < b4**** |

## Happiness

The mean values for each condition and robot type are shown in Figure 2. We conducted a two-factor repeated measures ANOVA, and the result showed a significant difference in main effects of addition of tears (*F*(1, 49) = 26.42, *p*<.001, *η*^2^ = 0.04), main effects of robot type (*F*(1.68, 82.21) = 86.26, *p*<.001, *η*^2^ = 0.34), and the interaction effects (*F*(2.6, 127.2) = 27.59, *p*<.001, *η*^2^ = 0.05). The results of the simple main-effect tests are presented in Table 9. The simple main effect of the addition of tears for all robot types was significant. Thus, the mean value of the Tears condition was significantly higher than that of the No tears condition. The effects of the robot-type simple main effects in the No tears and Tears conditions were also significant; therefore, tests of multiple comparisons were conducted (Table 10).

**Figure 7. Mean of emotional intensity of happiness for each condition and type of robot.** Error bars represent the standard error of the mean.

**Table 9. Results of the simple main effect test of happines**. A= The addition of tears factor, a1=No tears condition, a2=Tears condition, B= Robot type factor.

|  | **SS** | **df** | **MS** | ***F*** | ***p*** | **η^2^** |
| --- | --- | --- | --- | --- | --- | --- |
| **A at b1** | 12210.47 | 1 | 12210.47 | 31.98 | <.001 | 0.12 |
| **Error (A at b1)** | 18710.12 | 49 | 381.84 |  |  |  |
| **A at b2** | 0.19 | 1 | 0.18 | 0.002 | 0.96 | 0.000 |
| **Error (A at b2)** | 3681.75 | 49 | 75.14 |  |  |  |
| **A at b3** | 49.94 | 1 | 49.94 | 0.20 | 0.66 | 0.002 |
| **Error (A at b3)** | 12361.21 | 49 | 252.27 |  |  |  |
| **A at b4** | 19034.34 | 1 | 19034.34 | 42.46 | <.001 | 0.19 |
| **Error (A at b4)** | 21966.44 | 49 | 448.29 |  |  |  |
| **B at a1** | 114817.80 | 1.94 | 59085.53 | 110.13 | <.001 | 0.53 |
| **Error (B at a1)** | 51084.20 | 95.22 | 536.49 |  |  |  |
| **B at a2** | 24524.22 | 1.99 | 12339.12 | 24.86 | <.001 | 0.20 |
| **Error (B at a2)** | 48329.34 | 97.39 | 496.25 |  |  |  |

**Table 10. Results of multiple comparisons of happines.** Asterisks indicate significant differences (***p* <.001, ****p* <.001).

| **Robot type in the No tears condition** | | | | | | |
| --- | --- | --- | --- | --- | --- | --- |
| **Pair** | **diff** | ***t*** | ***df*** | ***p*** |  |  |
| **b1-b2** | 43.01 | 9.36 | 49 | <.001 |  | **b1 > b2***** |
| **b1-b3** | 40.83 | 9.60 | 49 | <.001 |  | **b1 > b3***** |
| **b1-b4** | -10.74 | 3.22 | 49 | 0.005 |  | **b1 < b4**** |
| **b2-b3** | -2.17 | 1.15 | 49 | 0.26 |  | **b2 = b3** |
| **b2-b4** | -53.75 | 13.75 | 49 | <.001 |  | **b2 < b4***** |
| **b3-b4** | -51.57 | 13.70 | 49 | <.001 |  | **b3 < b4***** |
|  | | | | | | |
| **Robot type in the Tears condition** | | | | | | |
| **Pair** | **diff** | ***t*** | ***df*** | ***p*** |  |  |
| **b1-b2** | 20.82 | 5.21 | 49 | <.001 |  | **b1 < b2***** |
| **b1-b3** | 17.32 | 4.64 | 49 | <.001 |  | **b1 < b3***** |
| **b1-b4** | -5.25 | 1.69 | 49 | 0.18 |  | **b1 = b4** |
| **b2-b3** | -3.50 | 1.74 | 49 | 0.18 |  | **b2 = b3** |
| **b2-b4** | -26.07 | 6.15 | 49 | <.001 |  | **b2 > b4***** |
| **b3-b4** | -22.67 | 5.42 | 49 | <.001 |  | **b3 > b4***** |

# Conclusion

These results suggest that there may be differences in the effects of tear addition between the different robot types. However, the effect of tears in enhancing sadness was confirmed for all robots and the effect size was high (η^2^ = 0.56 ~ 0.79).
